# Predicting Adverse Events During Six-Minute Walk Test Using Continuous Physiological Signals

**DOI:** 10.3389/fphys.2022.887954

**Published:** 2022-06-06

**Authors:** Jiachen Wang, Yaning Zang, Qian Wu, Yingjia She, Haoran Xu, Jian Zhang, Shan Cai, Yuzhu Li, Zhengbo Zhang

**Affiliations:** ^1^ Medical School of Chinese PLA, Beijing, China; ^2^ Department of Kinesiology, Shanghai University of Sport, Shanghai, China; ^3^ Department of Pulmonary and Critical Care Medicine, the Second Xiangya Hospital, Central South University, Changsha, China; ^4^ Department of Pulmonary and Critical Care Medicine, Hainan Hospital of PLA General Hospital, Sanya, China; ^5^ Center for Artificial Intelligence in Medicine, Chinese PLA General Hospital, Beijing, China

**Keywords:** 6-min walk test, adverse events, machine learning, wearable devices, physiological signals

## Abstract

**Background and Objective:** The 6-min walk test (6MWT) is a common functional assessment test, but adverse events during the test can be potentially dangerous and can lead to serious consequences and low quality of life. This study aimed to predict the occurrence of adverse events during 6MWT, using continuous physiological parameters combined with demographic variables.

**Methods:** 578 patients with respiratory disease who had performed standardized 6MWT with wearable devices from three hospitals were included in this study. Adverse events occurred in 73 patients (12.6%). ECG, respiratory signal, tri-axial acceleration signals, oxygen saturation, demographic variables and scales assessment were obtained. Feature extraction and selection of physiological signals were performed during 2-min resting and 1-min movement phases. 5-fold cross-validation was used to assess the machine learning models. The predictive ability of different models and scales was compared.

**Results:** Of the 16 features selected by the recursive feature elimination method, those related to blood oxygen were the most important and those related to heart rate were the most numerous. Light Gradient Boosting Machine (LightGBM) had the highest AUC of 0.874 ± 0.063 and the AUC of Logistic Regression was AUC of 0.869 ± 0.067. The mMRC (Modified Medical Research Council) scale and Borg scale had the lowest performance, with an AUC of 0.733 and 0.656 respectively.

**Conclusion:** It is feasible to predict the occurrence of adverse event during 6MWT using continuous physiological parameters combined with demographic variables. Wearable sensors/systems can be used for continuous physiological monitoring and provide additional tools for patient safety during 6MWT.

## Introduction

The 6-min walk test (6MWT) is a submaximal, simple, low-cost, and effective exercise test used to obtain the functional capacity of patients with moderate to severe cardiopulmonary disease (Solway et al., 2001; American Thoracic Society, 2002). The 6-min walk distance (6MWD) is one of the key observations in clinical trials, and can be used as a predictor of diagnosis, prognosis and survival of patients with cardiopulmonary diseases, such as chronic obstructive pulmonary disease (COPD) (Casanova et al., 2008; Dajczman et al., 2015; Meena et al., 2020), interstitial lung diseases (Brown and Nathan, 2018), pulmonary hypertension (Farber et al., 2015; Gadre et al., 2017) and lung transplant (Castleberry et al., 2017).

6MWT has been used as a part of the standard procedure for cardiopulmonary function assessment and is considered to be safe for most patients. However, given the complexity of respiratory disease and the severity of the disease, adverse events during the 6MWT are still potentially dangerous.

Jenkins and Cecins (2011) suggested to revise the American Thoracic Society (ATS) 6MWT guidelines to monitor SpO_2_ continuously during 6MWT, because oxygen desaturation may increase the possibility of cardiac or other complications. 3.9% of patients with acute myocardial infarction (AMI) suffered angina, drop in blood pressure, or ventricular tachycardia (Diniz et al., 2017). Roberts et al. (2015) reported that there were lower quality of life and mood scores among patients who experienced adverse events compared with patients without adverse events. Serious adverse events may lead to the death of inpatients, the occurrence of life-threatening events, or the extension of the current hospital stay (Morris et al., 2015). Therefore, it is particularly important to be able to predict adverse events simply and quickly. In addition, since 6MWT is closer to daily life than lung function tests to assess the patient’s overall performance, the prediction of adverse events in 6MWT is also instructive for early warning in routine daily monitoring.

However, traditionally, the occurrence of adverse events is mainly judged by medical staff’s observation on patient’s performance and the patient’s complaint. Moreover, ATS guidelines (2002) and some studies (Roberts et al., 2015; Douwes et al., 2016) only used oximeters to monitoring oxygen saturation intermittently. This type of subjective judgments and short-term data monitoring of physiological parameters are not very useful for rapid identification of adverse events. Variation in physiology parameters can reflect changes in the state of the human body, even hours before an adverse event occurs (Brekke et al., 2019; Leenen et al., 2020; Yilmaz et al., 2020). Rodríguez et al. (2017) reported that heart rate recovery at 1 min (HRR1) after 6MWT is an independent predictor factor for acute exacerbation of COPD (AECOPD). Morita et al. (2018) reported that HRR1 may reflect the patient’s exercise capacity, lifestyle and functional status. Mazzuco et al. (2017) reported that heart rate variability (HRV) can be useful in the functional assessment of COPD. The emergence of wearable physiological parameter monitoring systems provides the technical means for continuous and non-invasive dynamic monitoring of physiological parameters during 6MWT.

In order to reduce the risk of patients during 6MWT and remind nurses to pay attention to high-risk groups, this study aimed to predict the occurrence of adverse events during 6MWT through continuous dynamic physiological parameters monitored by wearable devices.

## Methods

### Patients

The multi-center study was conducted at Chinese PLA General Hospital, Hainan Hospital of Chinese PLA General Hospital and the Second Xiangya Hospital of Central South University. Patients with respiratory disease who undertook 6MWT with wearable devices from June 2019 to September 2020 were included. Exclusion criteria: 1) patients with comorbid neurological and muscular diseases or limited daily activities. 2) Patients with missing or poor-quality signals and those who did not complete 6MWT as required by the protocol. 3) Patients with unstable angina or myocardial infarction within 1 month. The study was approved by the Medical Ethics Committee (Ethics No.: S2018-095-01, Clinical Trail No.: ChiCTR-POC-17010431).

578 patients were included in this study. Patients’ characteristics are shown in Table 1. The mean age was 62.2 ± 11.1 years old. The mean BMI was 23.1 ± 3.9. There were 39.4% of the patients who had COPD, the most common disease among the patients. 81 patients were combined with hypertension. 38 patients were combined with coronary heart disease. 31 patients were combined with diabetes. The mean score on Borg fatigue score was 0.6 ± 1.0. The mean score on modified Medical Research Council (mMRC) dyspnea score was 1.2 ± 1.3.

**TABLE 1:** Baseline demographic data.

Variable	Value
Demographics	
Number	578
Gender (male), *n* (%)	435 (75.3)
Age (years), mean (SD)	62.2 (11.1)
Height (m), mean (SD)	163 (6.94)
Weight (kg), mean (SD)	61.7 (12.4)
BMI, mean (SD)	23.1 (3.9)
6MWD (m), mean (SD)	413.3 (101.3)
Principal Diagnosis, *n* (%)	
COPD	228 (39.4)
Pneumonia	48 (8.3)
Bronchiectasis	77 (13.3)
Asthma	76 (13.1)
Lung cancer	59 (10.2)
Pulmonary fibrosis	52 (9.0)
Pulmonary arterial hypertension	24 (4.2)
Pulmonary embolism	14 (2.4)
Scales, mean (SD)	
Borg	0.6 (1.0)
mMRC	1.2 (1.3)
Adverse events, *n* (%)	
Intolerable dyspnea	66 (11.4)
Chest pain or tightness	17 (2.9)
Lack of physical strength	14 (2.9)
Palpitation	10 (1.7)
Dizziness	5 (0.9)
Oxygen inhalation	5 (0.9)

BMI, body mass index; 6MWD, 6-min walk distance; COPD, chronic obstructive pulmonary disease; IPF, idiopathic pulmonary fibrosis; mMRC, modified medical research council.

### Multi-Functional 6-Min Walk Test System

**FIGURE 1:**
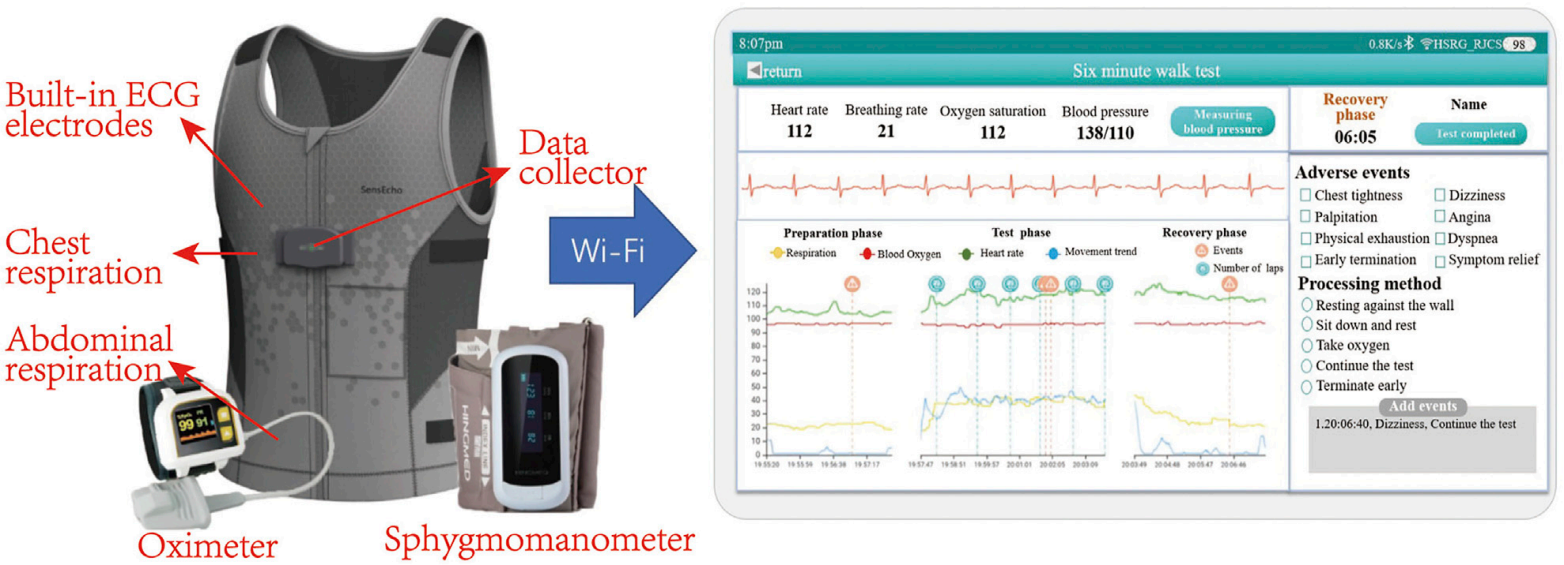
Picture of multi-functional 6MWT system, including wearable devices and an intelligence terminal device (a hand-held PAD). Wearable devices include a flexible vest, an oximeter and a blood pressure monitor. The system is able to monitor ECG, respiratory waves, triaxial acceleration, oxygen saturation and blood pressure. The physiological signal can be transmitted to the PAD *via* Wi-Fi and displayed in real time.

In this study, data were collected by using a sensor-based system, which achieves physiological signal monitoring and recording during the whole process of 6MWT. The system consists of two key components as shown in Figure 1: the flexible vest and the intelligence terminal device (i.e., a hand-held PAD). The flexible vest is our self-developed wearable device, SensEcho, which is able to provide ECG, respiratory waves and triaxial acceleration signals, and to communicate with third-party devices such as blood pressure monitor and oximeter. The flexible vest is connected with ECG electrodes, which can collect single-lead ECG signal. Two elastic bands are embedded in the chest and abdomen positions respectively using respiratory induction plethysmography (RIP) technology, which allow accurate recording of chest and abdominal breathing movement. The triaxial acceleration sensor is built into the data collector. Oxygen saturation and blood pressure were measured by oximeter and sphygmomanometer respectively.

The real-time physiological signals can be displayed on the terminal device, and the system provides the functions of early warning based on physiological signal monitoring and manually recording the adverse events, such as hypoxia or dyspnea. Moreover, the system is able to generate a report which summarizes the performance of the patient in the 6MWT by comprehensive analysis of physiological parameters and upload the overall data of 6MWT process to the cloud sever.

### 6-Min Walk Test Protocol

All hospitals followed the same 6MWT protocol. About 10 min before the 6MWT, the patient put on the SensEcho device and had a rest. Medical staff opened the application on the PAD and connected the terminal to measure basic physiological parameters such as heart rate, breath rate, blood pressure and oxygen saturation remotely. Meanwhile, patients were instructed to complete Borg fatigue score, mMRC dyspnea scale on the PAD.

The 6MWT was carried out in a 30-m-long flat corridor in accordance with the recommended ATS scheme. Once the patient started walking on the start line, a timer was activated and patients should walk as far as possible for 6 min. At 1-min intervals, the app on the pad encouraged the patient in a standardized way. Medical staff observed the patient’s ECG, respiration, and oxygen saturation on the PAD in real time and recorded the laps. If an adverse event occurred during the 6MWT, the event and the treatment would be recorded on the PAD in time. Patients can have a rest or stop the test at any time. At the end of 6MWT, patients would be asked to stop and rest against the wall for 2 min before taking off the device and 6MWD was recorded.

### Adverse Events

According to ATS guidelines and previous studies, symptoms such as intolerable dyspnea, palpitation, dizziness, chest pain or chest tightness, fatigue and hypoxia during 6MWT were considered as adverse events (American Thoracic Society, 2002; Jenkins and Cecins, 2011; Roberts et al., 2015).

**FIGURE 2:**
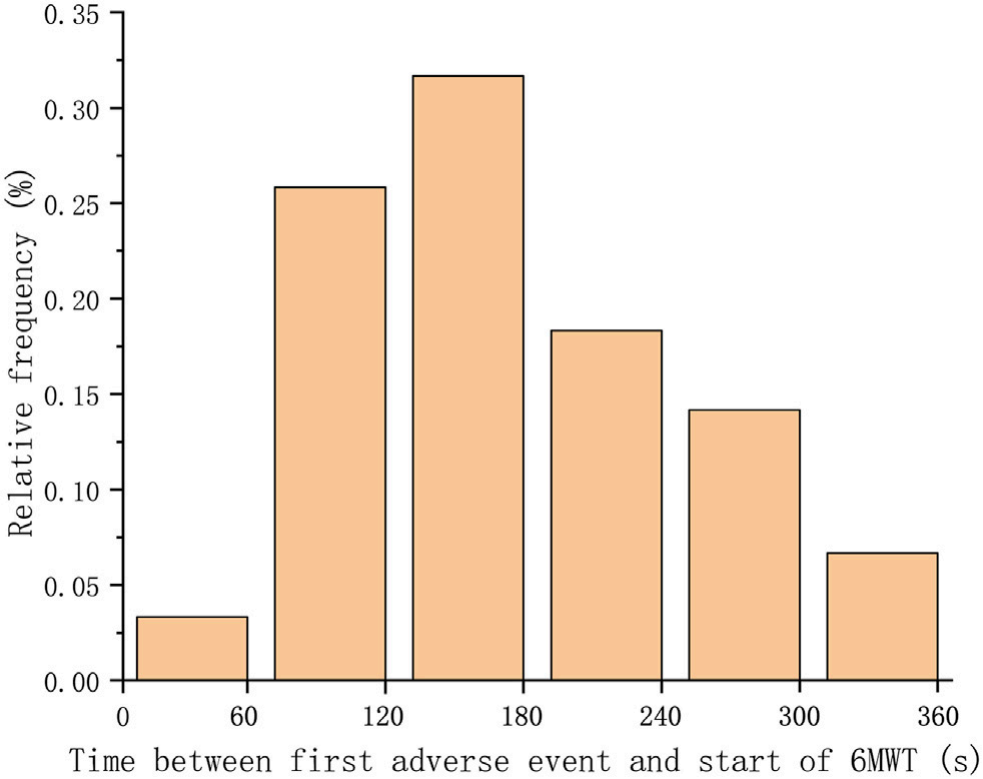
Distribution of the time between the occurrence of the first adverse event and the beginning of the 6MWT. Adverse events of over 95% of patients occurred after 1 min.

73 patients (12.6% of total cohort) experienced adverse events. 10 patients have prematurely terminated the test due to intolerable dyspnea or oxygen therapy. As shown in Table 1, the most common adverse event was intolerable dyspnea (66 patients). The mean distance of all patients was 413.3 ± 101.3 m. The mean distance walked by patients who developed adverse event was 278.1 ± 132.1 m. The mean distance walked by patients without adverse events was 444.0 ± 90.9 m. When a patient had more than one adverse event during 6MWT, we adopted the time of the first occurrence. The time distribution of adverse events among patients was shown in Figure 2. It could be seen that approximately 95% adverse events occurred 1 min after the beginning of 6MWT. Only four patients had an adverse event within 1 min and the adverse event they had was intolerable dyspnea.

### Data Pre-Processing and Feature Extraction

Firstly, the raw ECG and respiratory signals were smoothed and filtered and outliers were removed. Then, Hamilton’s method (Hamiltom and Tompkins, 1986) was used to detect R peaks of ECG signal. Khodadad’s method (Khodadad et al., 2018) was used to detect peaks and valley value of respiratory signal.

55 features were divided into five categories, including ECG signal features, respiratory signal features, SpO_2_ features, motion amplitude features and demographic features. We selected resting segment data of the 2 min before 6MWT to calculated the average resting heart rate (HR_rest_), respiratory rate (RR_rest_), SpO_2_ (SpO_2 rest_) and HRV parameters (time-domain parameters, frequency domain parameters and nonlinear-domain parameters). Few adverse events occurred in the first minute, so the first minute data during 6MWT was included to calculate features. The peak heart rate (HR_peak_), peak respiratory rate (RR_peak_), minimum SpO_2_ (SpO_2 min_) and mean heart rate (HR_mean_), respiratory rate (RR_mean_) and SpO_2_ (SpO_2 mean_) during the first minute of 6MWT were calculated. Then, the difference between corresponding features in resting phase and 6MWT process was calculated.

In addition, HR slope and HR intercept were respectively defined as the slope and intercept of line that fits the beat-by-beat HR of the first minute 6MWT process by the least square method. The oxygen desaturation area was defined as the sum of the difference between the baseline of oxygen saturation and the oxygen saturation per second during the 6MWT process (Flaherty et al., 2006). Demographic features included age, gender and BMI. Motion amplitude feature, as shown in following, was calculated by the triaxial acceleration signal, transforming the raw *x*, *y*, and *z* acceleration data into signal vector magnitude (SVM) data (Bidargaddi et al., 2007).

$$\text{SVM}=\sqrt{\overset{n}{\underset{k=0}{\sum }}\left(X_{k}^{2}+Y_{k}^{2}+Z_{k}^{2}\right)}$$

In the above formula*, n* is the product of the sampling rate and the recording time. $X_{k}$ is a vector representing the acceleration along X axis, while the other axes are represented by vectors of $Y_{k}$ , and $Z_{k}$ , respectively.

### Models

First, the recursive feature elimination method based on Logistic Regression with L1 and L2 regularization was used to select the best features to prevent overfitting. Then, 5-fold cross validation was used to train and test the models including Logistic Regression, Support Vector Classifications (SVCs), Random Forest, eXtreme Gradient Boosting (XGBoost) and Light Gradient Boosting Machine (LightGBM) with the selected features. For comparison, we also calculated scales’ ability to predict adverse events, including mMRC, Borg before 6MWT. The predictive ability of the models and scales were evaluated through ROC curve and area under the curve (AUC). In addition, sensitivity, specificity, positive likelihood ratio and negative likelihood were also calculated. Comparisons among machine learning models were provided as well.

## Results

### Adverse Events

**FIGURE 3:**
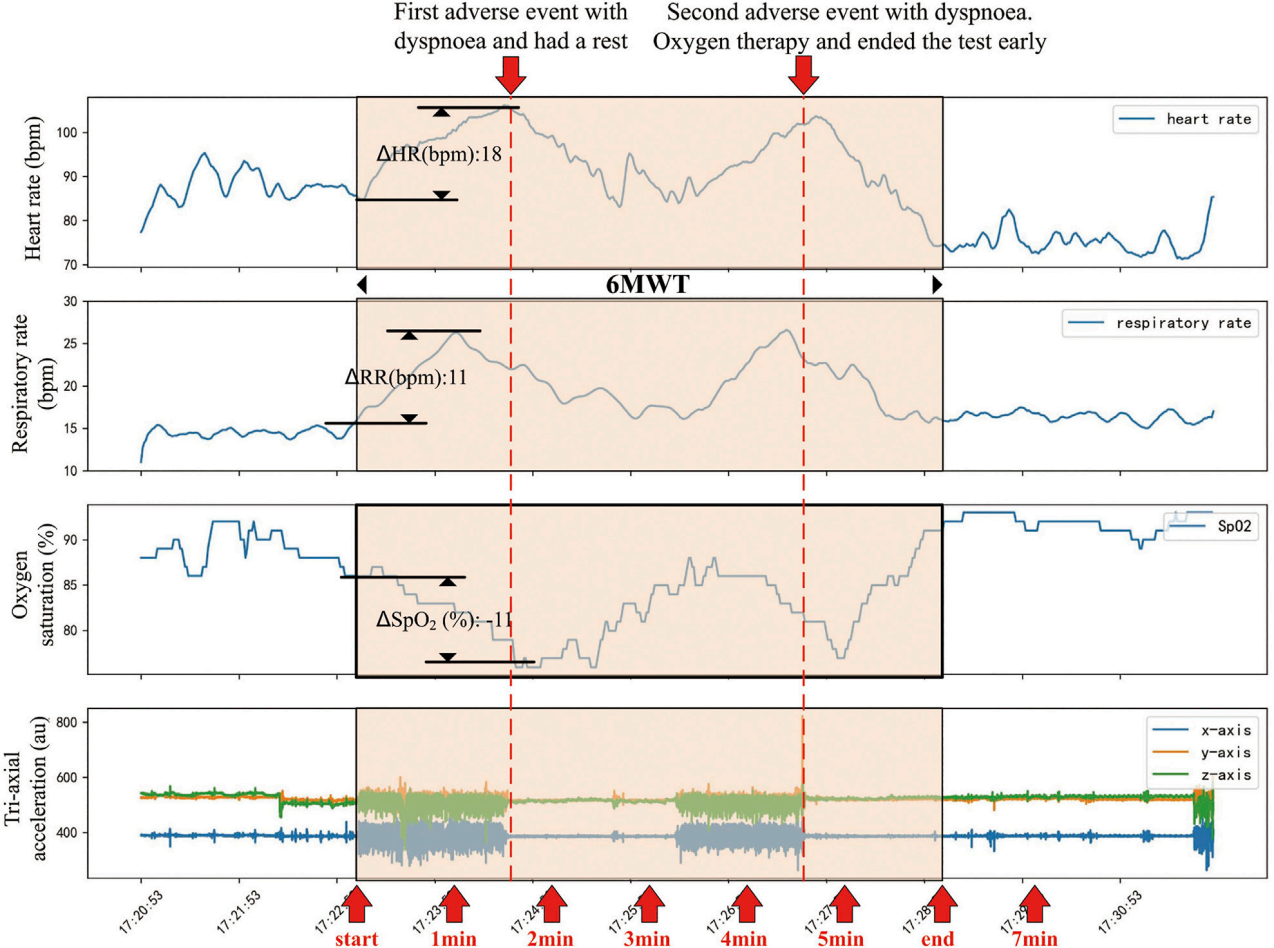
An example of changes in physiological parameters of a patient before, during and after the 6MWT process. This patient had a rapid increase in heart rate and respiratory rate while a rapid drop in oxygen saturation following the beginning of 6MWT. About 90 s after the beginning of 6MWT, the patient had a dyspnea and had a rest. During the rest period, the patient’s heart rate, respiratory rate and oxygen saturation recovered. About 280 s after the beginning of 6MWT, the patient developed a more severe intolerable dyspnea and was treated with oxygen. The 6MWT was terminated about 80 s early.

Figure 3 showed the trend of physiological signals and the occurrence of adverse events in a patient during the 6MWT process. When the patient started walking, the heart rate and breath rate increased, while oxygen saturation dropped rapidly. There were two adverse events during the 6MWT, the second of which was severe intolerable dyspnea and the patient received oxygen therapy after that.

### Model Performance

Combining the characteristics of the resting phase and the movement phase, the AUC reached the maximum value when 16 features were selected.

**FIGURE 4:**
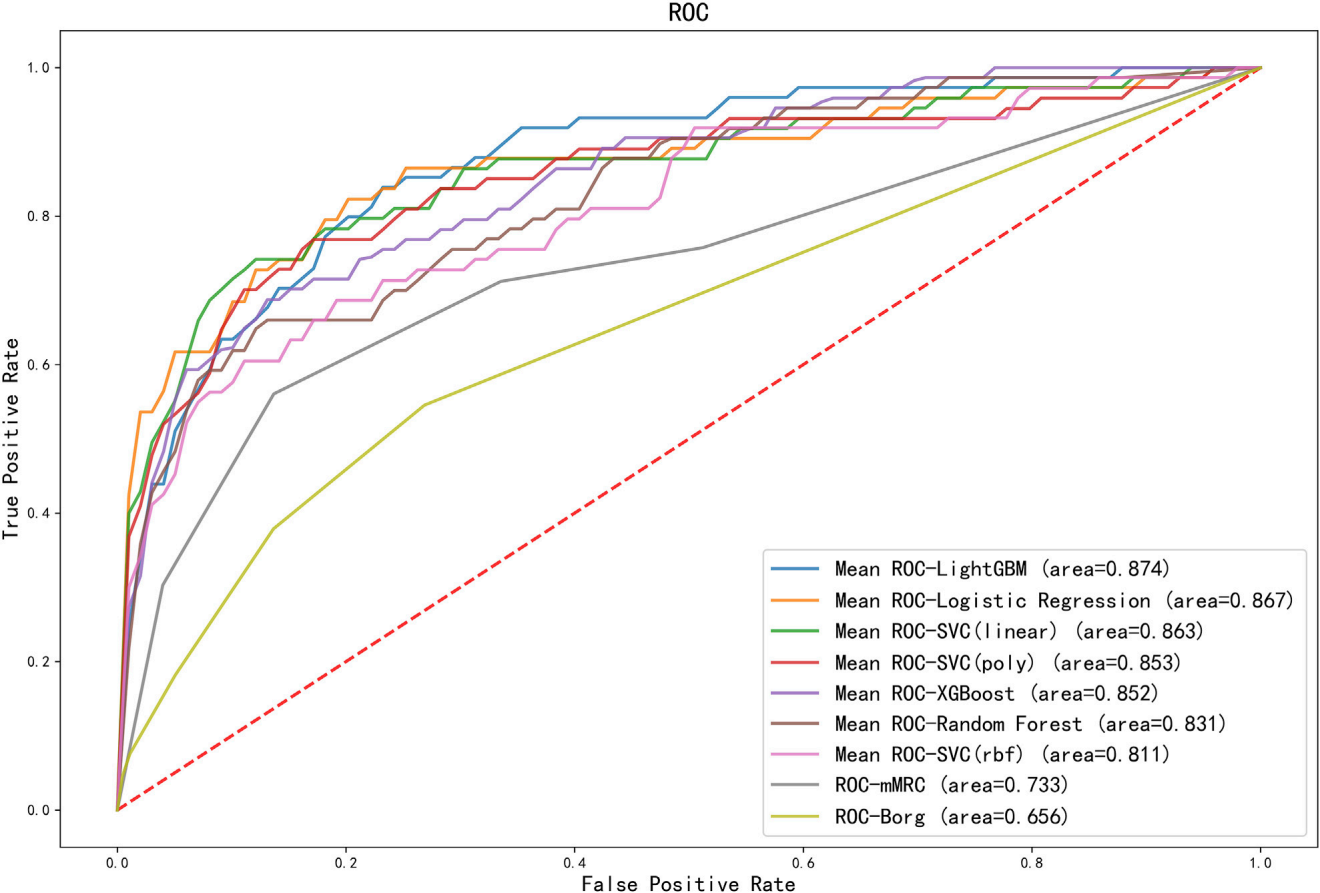
ROC curves for different machine learning models and scoring scales. The ROC curves for machine learning models were the average score of the results of a five-fold cross-validation. AUC results of all machine learning models outperformed the scales. SVC, support vector classification; LightGBM, light gradient boosting machine; XGBoost, extreme gradient boosting; mMRC, modified medical research council dyspnea scale.

The ROC curves comparing machine learning models using Logistic Regression, SVCs, Random Forest, LightGBM and XGBoost and scales using mMRC and Borg were presented in Figure 4. The ROC curve was the average result of 5-fold cross-validation. As shown in Figure 4, machine learning models performed better than traditional scales. Among models, the machine learning model with the best performance was LightGBM, with a mean AUC of 0.874, while the mMRC scale and Borg scale had the lowest performance, with an AUC of 0.733 and 0.656 respectively.

**FIGURE 5:**
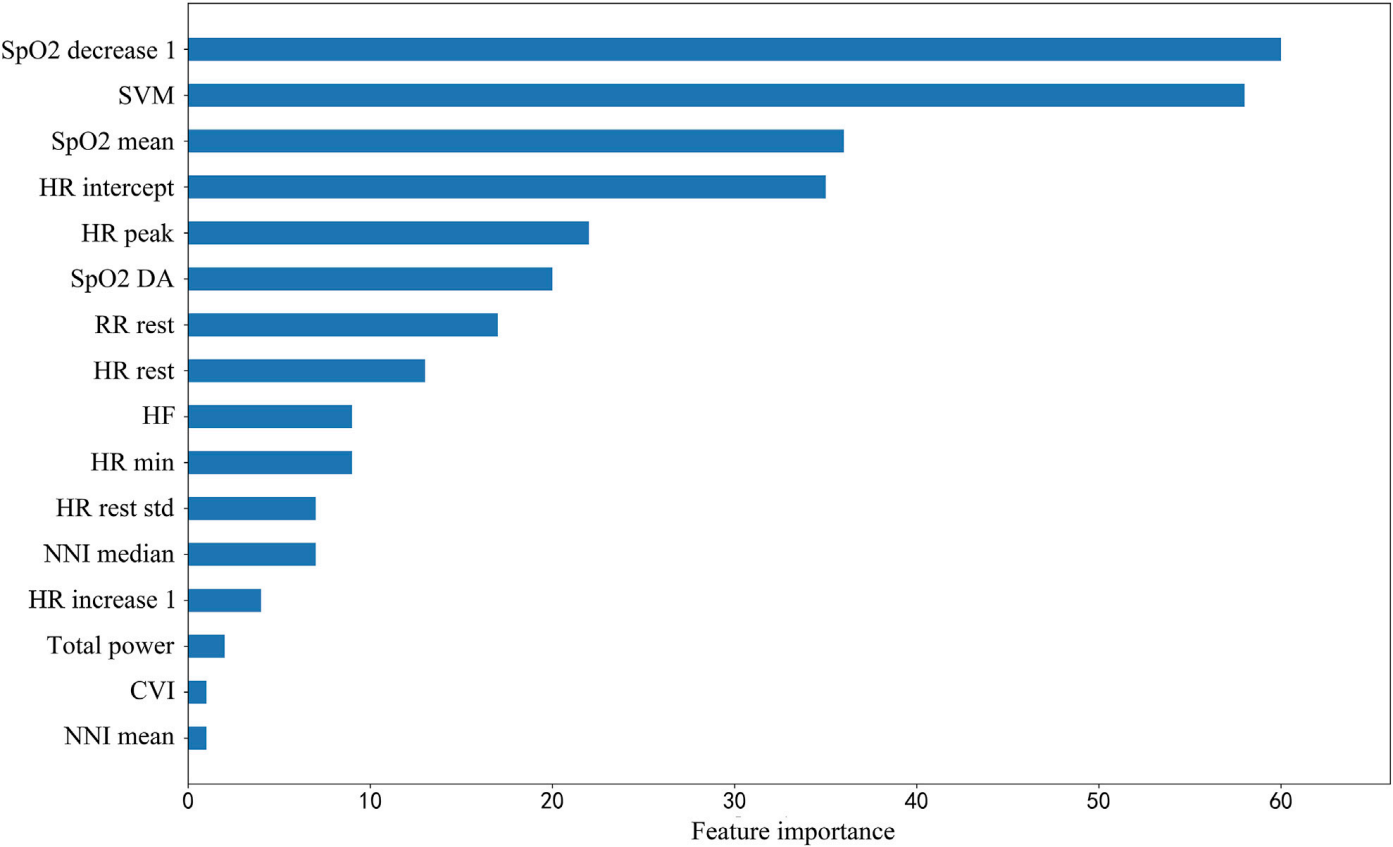
Feature importance ranking in lightGBM model. SpO2_decrease1, Spo2 decrease value in 1 min; SVM, signal vector magnitude; SpO2 mean 1, mean value of SpO2 in first 1 min; HR intercept, heart rate intercept; HR peak, peak heart rate; SpO2 DA, SpO2 desaturation area; RR rest, breath rate during rest segment; HR rest, heart rate during rest segment; HF, high frequency power, one of HRV parameters; HR min, minimum heart rate; HR rest std, standard deviation of beat-to-beat heart rate during rest segment; HR increase 1, heart rate increase value in 1 min; Total power, one of HRV parameters; CVI, cardiac vagal index, one of HRV parameters; NNI mean, mean value of normal-to-normal intervals.

The classification results comparing different machine learning models using 5-fold cross-validation were presented in Table 2. The performance of LightGBM was stable and the best among machine learning models, with the highest AUC and the smallest standard deviation. The model with the highest positive likelihood ratio was SVC (linear kernel), and the model with the lowest negative likelihood ratio was LightGBM. Feature importance ranking in lightGBM model was shown in Figure 5.

**TABLE 2:** Comparison between different machine learning models validated by 5-fold validation.

	AUC	SN	SP	+LR	−LR
LightGBM	0.874 ± 0.063	0.859	0.768	3.616	0.184
Logistic Regression	0.868 ± 0.067	0.837	0.778	3.770	0.210
SVC (linear)	0.863 ± 0.070	0.742	0.879	6.132	0.294
SVC (poly)	0.853 ± 0.085	0.769	0.829	4.497	0.279
XGBoost	0.852 ± 0.071	0.688	0.869	5.252	0.359
Random forest	0.831 ± 0.070	0.66	0.869	5.038	0.391
SVC (rbf)	0.811 ± 0.098	0.687	0.808	3.578	0.387

AUC, area under the curve; SN, sensitivity; SP, specificity; +LR, positive likelihood ratio; −LR, negative likelihood ratio.

## Discussion

The multifactorial complexity of diseases and the human body makes it challenging to predict adverse events during 6MWT. As far as we know, many studies have focused on the prediction of 6MWD and the relationship between 6MWD and diseases. However, there are few studies predicting adverse events with continuous physiological parameters combined with easily measurable demographic variables, possibly due to a lack of powerful tools. The combination of wearable physiological parameter monitoring devices and interpretable artificial intelligence can help solve these problems. Machine learning algorithms are able to recognize patterns within data. A data model built on the learned patterns from the training dataset predicts the results of the unseen test dataset. Similar to a human learning process, machine learning algorithms become more powerful with more increased data and experience. This study provides an assistive decision support tool for whether patients need additional medical help during 6MWT. At the same time, this study also provides new ideas and methods assessing the safety and security of patients during 6MWT.

High percentage of adverse events occurrence from the first minute is an objective fact found in the large sample of this study. As for the reasons, the 6MWT is a sub-maximal exercise test that assesses functional capacity by measuring the total distance walked in 6 min. The activity level in the first minute may not be enough to cause adverse events. On the other hand, changes in physiological parameters may precede the onset of symptoms (Brekke et al., 2019).

Scales are the most frequently used method to assess the patients’ physical condition. However, the scale method is easily affected by individual subjectivity. Physiological parameters can be used for objective and quantitative assessment of physical state. As shown in Figure 3, physiological parameters changed significantly before and after 6MWT and adverse events. As shown in Figure 4, the predictive ability of the combination of machine learning and physiological parameters was significantly better than the predictive ability of scales. To further improve the 6MWT assessment, a physiological parameters-based assessment scale similar to the NEWS score (national early warning score, to identify patients with high risk of an acute and unstable disease) could be established as a later research direction.

Among the selected features, heart rate related features and HRV features are the most common ones, which are often ignored in clinical researches. Heart rate and HRV are regulated by sympathetic nerves and parasympathetic nerves, which is a reliable reflection of the many physiological factors modulating the normal rhythm of the heart. Therefore, heart rate and HRV can reflect changes in the body’s state more accurately (Acharya et al., 2006). Moreover, the resting heart rate is relatively low in people with better cardiopulmonary function, so HR_rest_ is also an important indicator for predicting adverse events. The increase in heart rate within 1 min represents the heart’s ability to respond to exercise (Cannière et al., 2020). HR intercept, another expression of HR_rest_, has the advantage of being less affected by abnormal values. As for HRV, total power density spectral is an important predictor. High-frequency HRV reflects rapid changes in beat-to-beat variability caused by the activity of parasympathetic nerve (vagal nerve) (Ziemssen and Siepmann, 2019). Patients with chronic respiratory disease, especially those with COPD and idiopathic pulmonary fibrosis (IPF), have an increased respiratory rate (Schertel et al., 2017; McKinstry et al., 2018). Therefore, RR_rest_ is also a reflection of cardiopulmonary function. To our surprise, the change in respiratory rate during walking is not a significant predictor. Although studies have shown that asymptomatic exertional hypoxemia is not associated with an increase in the incidence of complications or adverse events during the 6MWT (Roberts et al., 2015; Afzal et al., 2018), in our study, the decreased oxygen saturation values and the oxygen desaturation area are still important for predicting adverse events. The SVM is an indicator to measure the intensity of physical activity, and it reflects the exercise performance during 6MWT. Patients with poor exercise capacity are more likely to have adverse events.

Currently, 6MWT is widely used in clinical practice. However, the entire 6MWT process presents the following risks and shortcomings. First, 6MWT is mostly performed in patients who are post-surgical or have severe cardiopulmonary disease, which poses the test a certain risk. This risk will increase due to the lack of objective monitoring throughout the test (Morris et al., 2015). Second, with the highly developed information technology, evaluation of a patient’s cardiopulmonary function by a single indicator (6MWD) will inevitably leave out a large amount of high-value information. Moreover, the 6MWD is extremely sensitive to methodology (Agarwala and Salzman, 2020) and the same 6MWD evaluation approach and grading criteria may not be applicable in the different test site and population (Dourado, 2011). It is reported that differences in the performance of individuals on the 6MWT within Brazil and abroad still existed, and it is necessary to provide specific calculations and evaluation methods for each population and ethnic group (Dourado, 2011). The sensor-based 6MWT is therefore particularly important. The appearance of wearable physiological monitoring systems has greatly facilitated the application of 6MWT, ensuring the safety of the test and making full use of the data. In addition, the sensor-based 6MWT offers patients a personalized approach to cardiopulmonary function assessment.

An advantage of wearable devices is that they can provide mobile and ubiquitous health monitoring in daily life. The model proposed in this paper can be applied to daily life monitoring, especially to identify people at high risk of adverse events because their quality of life and mood scores are worse (Roberts et al., 2015). In addition to adverse events during 6MWT, there are few studies on the association of physiological parameters during 6MWT with adverse events in other scenarios. For example, postoperative pulmonary complications (PPCs) are significant causes of increased length of stay and medical costs, poor prognosis, and death in patients undergoing heart valve surgery. Although 6MWT results have been shown to be associated with adverse prognostic events and risk of death (Casanova et al., 2008), its application to predict PPCs has not been reported. This study has a high reference value for this direction.

There are still some limitations in this study. Firstly, in order to simplify the models, improve the practicality and reduce the burden on healthcare professionals, the patient’s spirometry values, comorbidities, lab tests and medication were not included in the model and it can be expected that the inclusion of the above parameters would help to further improve the model performance. Secondly, the diseases of the patients included in this study were complex and the probability of adverse events varied from patients to patients with different diseases. However, from another perspective, the complexity of the disease in multi-center patients made the models had a certain degree of extrapolation. Thirdly, due to the sample size, we treated adverse events as an overall outcome. In the follow-up study, we will expand the sample size and focus on each outcome independently. Meanwhile, with the increased sample size, the performance of the model will also be improved and robust.

Adverse events during 6MWT can be dangerous. Our study predicted the occurrence of adverse events using continuous physiological parameters collected by the sensor-based 6MWT system from multi-center patients during the 6MWT. This study provides additional safety for the patients and offers new methods and ideas for patient monitoring during 6MWT.

## Data Availability

The datasets presented in this article are not readily available because patient privacy needs to be protected. Requests to access the datasets should be directed to the corresponding author.

## References

[B1] Afzal S. Burge A. T. Lee A. L. Bondarenko J. Holland A. E. (2018). Should the 6-Minute Walk Test Be Stopped if Oxyhemoglobin Saturation Falls below 80%? Arch. Phys. Med. Rehabil. 99, 2370–2372. 10.1016/j.apmr.2018.07.426 30130517

[B2] Agarwala P. Salzman S. H. (2020). Six-Minute Walk Test. Chest 157, 603–611. 10.1016/j.chest.2019.10.014 31689414 PMC7609960

[B3] American Thoracic Society (2002). ATS Statement: Guidelines for the Six-Minute Walk Test. Am. J. Respir. Crit. Care Med. 166, 111–117. 10.1164/ajrccm.166.1.at1102 12091180

[B4] Bidargaddi N. Sarela A. Klingbeil L. Karunanithi M. (2007). “Detecting Walking Activity in Cardiac Rehabilitation by Using Accelerometer,” in 2007 3rd International Conference on Intelligent Sensors (Sensor Networks and Information), 555–560. 10.1109/ISSNIP.2007.4496903

[B5] Brekke I. J. Puntervoll L. H. Pedersen P. B. Kellett J. Brabrand M. Patman S. (2019). The Value of Vital Sign Trends in Predicting and Monitoring Clinical Deterioration: A Systematic Review. PLoS ONE 14, e0210875. 10.1371/journal.pone.0210875 30645637 PMC6333367

[B6] Brown A. W. Nathan S. D. (2018). The Value and Application of the 6-Minute-Walk Test in Idiopathic Pulmonary Fibrosis. Ann. ATS 15, 3–10. 10.1513/AnnalsATS.201703-244FR 28933948

[B7] Casanova C. Cote C. Marin J. M. Pinto-Plata V. De Torres J. P. Aguirre-Jaíme A. (2008). Distance and Oxygen Desaturation during the 6-min Walk Test as Predictors of Long-Term Mortality in Patients with COPD. Chest 134, 746–752. 10.1378/chest.08-0520 18625667

[B8] Castleberry A. Mulvihill M. S. Yerokun B. A. Gulack B. C. Englum B. Snyder L. (2017). The Utility of 6-minute Walk Distance in Predicting Waitlist Mortality for Lung Transplant Candidates. J. Heart Lung Transplant. 36, 780–786. 10.1016/j.healun.2016.12.015 28131666 PMC5495471

[B9] Dajczman E. Wardini R. Kasymjanova G. Préfontaine D. Baltzan M. A. Wolkove N. (2015). Six Minute Walk Distance Is a Predictor of Survival in Patients with Chronic Obstructive Pulmonary Disease Undergoing Pulmonary Rehabilitation. Can. Respir. J. 22, 225–229. 10.1155/2015/280187 26252533 PMC4530856

[B10] De Cannière H. Corradi F. Smeets C. J. P. Schoutteten M. Varon C. Van Hoof C. (2020). Wearable Monitoring and Interpretable Machine Learning Can Objectively Track Progression in Patients during Cardiac Rehabilitation. Sensors 20, 3601. 10.3390/s20123601 PMC7349532 32604829

[B11] Diniz L. S. Neves V. R. Starke A. C. Barbosa M. P. T. Britto R. R. Ribeiro A. L. P. (2017). Safety of Early Performance of the Six-Minute Walk Test Following Acute Myocardial Infarction: a Cross-Sectional Study. Braz. J. Phys. Ther. 21, 167–174. 10.1016/j.bjpt.2017.03.013 28473280 PMC5537468

[B12] Dourado V. Z. (2011). Equações de referência para o teste de caminhada de seis minutos em indivíduos saudáveis. Arq. Bras. Cardiol. 96, e128–e138. 10.1590/s0066-782x2011005000024 21359481

[B13] Douwes J. M. Hegeman A. K. Van Der Krieke M. B. Roofthooft M. T. R. Hillege H. L. Berger R. M. F. (2016). Six-minute Walking Distance and Decrease in Oxygen Saturation during the Six-Minute Walk Test in Pediatric Pulmonary Arterial Hypertension. Int. J. Cardiol. 202, 34–39. 10.1016/j.ijcard.2015.08.155 26386916

[B14] Farber H. W. Miller D. P. Mcgoon M. D. Frost A. E. Benton W. W. Benza R. L. (2015). Predicting Outcomes in Pulmonary Arterial Hypertension Based on the 6-minute Walk Distance. J. Heart Lung Transplant. 34, 362–368. 10.1016/j.healun.2014.08.020 25312386

[B15] Flaherty K. R. Andrei A.-C. Murray S. Fraley C. Colby T. V. Travis W. D. (2006). Idiopathic Pulmonary Fibrosis. Am. J. Respir. Crit. Care Med. 174, 803–809. 10.1164/rccm.200604-488OC 16825656 PMC2648064

[B16] Gadre A. Ghattas C. Han X. Wang X. Minai O. Highland K. B. (2017). Six-Minute Walk Test as a Predictor of Diagnosis, Disease Severity, and Clinical Outcomes in Scleroderma-Associated Pulmonary Hypertension: The DIBOSA Study. Lung 195, 529–536. 10.1007/s00408-017-0034-1 28646245

[B17] Hamilton P. S. Tompkins W. J. (1986). Quantitative Investigation of QRS Detection Rules Using the MIT/BIH Arrhythmia Database. IEEE Trans. Biomed. Eng. BME-33, 1157–1165. 10.1109/tbme.1986.325695 3817849

[B18] Jenkins S. Čečins N. (2011). Six-minute Walk Test: Observed Adverse Events and Oxygen Desaturation in a Large Cohort of Patients with Chronic Lung Disease. Intern Med. J. 41, 416–422. 10.1111/j.1445-5994.2010.02169.x 20059599

[B19] Khodadad D. Nordebo S. Müller B. Waldmann A. Yerworth R. Becher T. (2018). Optimized Breath Detection Algorithm in Electrical Impedance Tomography. Physiol. Meas. 39, 094001. 10.1088/1361-6579/aad7e6 30074906

[B20] Kütmeç Yilmaz C. Duru Aşiret G. Çetinkaya F. (2021). The Effect of Back Massage on Physiological Parameters, Dyspnoea, and Anxiety in Patients with Chronic Obstructive Pulmonary Disease in the Intensive Care Unit: A Randomised Clinical Trial. Intensive Crit. Care Nurs. 63, 102962102962. 10.1016/j.iccn.2020.102962 33162314

[B21] Leenen J. P. L. Leerentveld C. Van Dijk J. D. Van Westreenen H. L. Schoonhoven L. Patijn G. A. (2020). Current Evidence for Continuous Vital Signs Monitoring by Wearable Wireless Devices in Hospitalized Adults: Systematic Review. J. Med. Internet Res. 22, e18636. 10.2196/18636 32469323 PMC7351263

[B22] Mazzuco A. Medeiros W. M. Souza A. S. d. Alencar M. C. N. Neder J. A. Borghi-Silva A. (2017). Are Heart Rate Dynamics in the Transition from Rest to Submaximal Exercise Related to Maximal Cardiorespiratory Responses in COPD? Braz. J. Phys. Ther. 21, 251–258. 10.1016/j.bjpt.2017.05.002 28558953 PMC5537469

[B23] McKinstry S. Pilcher J. Bardsley G. Berry J. Van De Hei S. Braithwaite I. (2018). Nasal High Flow Therapy and PtCO2in Stable COPD: A Randomized Controlled Cross-Over Trial. Respirology 23, 378–384. 10.1111/resp.13185 28940962

[B24] Meena M. Singh R. Singh A. Motapothula U. (2020). Correlation of 6-Minute-Walk Test with Functional Profile in Stable Patients with Copd. Chest 158, A1765. 10.1016/j.chest.2020.08.1543

[B25] Morita A. A. Silva L. K. O. Bisca G. W. Oliveira J. M. Hernandes N. A. Pitta F. (2018). Heart Rate Recovery, Physical Activity Level, and Functional Status in Subjects with COPD. Respir. Care 63, 1002–1008. 10.4187/respcare.05918 29765005

[B26] Morris N. R. Seale H. Harris J. Hall K. Hopkins P. Kermeen F. (2015). Serious Adverse Events during a 6-min Walk Test in Patients with Pulmonary Hypertension. Eur. Respir. J. 45, 1179–1182. 10.1183/09031936.00146914 25614170

[B27] Rajendra Acharya U. Paul Joseph K. Kannathal N. Lim C. M. Suri J. S. (2006). Heart Rate Variability: a Review. Med. Bio Eng. Comput. 44, 1031–1051. 10.1007/s11517-006-0119-0 17111118

[B28] Roberts M. M. Cho J.-G. Sandoz J. S. Wheatley J. R. (2015). Oxygen Desaturation and Adverse Events during 6-min Walk Testing in Patients with COPD. Respirology 20, 419–425. 10.1111/resp.12471 25601398

[B29] Rodríguez D. A. Kortianou E. A. Alison J. A. Casas A. Giavedoni S. Barberan-Garcia A. (2017). Heart Rate Recovery after 6-min Walking Test Predicts Acute Exacerbation in COPD. Lung 195, 463–467. 10.1007/s00408-017-0027-0 28624883

[B30] Schertel A. Funke-Chambour M. Geiser T. Brill A.-K. (2017). Novel Insights in Cough and Breathing Patterns of Patients with Idiopathic Pulmonary Fibrosis Performing Repeated 24-Hour-Respiratory Polygraphies. Respir. Res. 18, 190. 10.1186/s12931-017-0674-y 29132424 PMC5683431

[B31] Solway S. Brooks D. Lacasse Y. Thomas S. (2001). A Qualitative Systematic Overview of the Measurement Properties of Functional Walk Tests Used in the Cardiorespiratory Domain. Chest 119, 256–270. 10.1378/chest.119.1.256 11157613

[B32] Ziemssen T. Siepmann T. (2019). The Investigation of the Cardiovascular and Sudomotor Autonomic Nervous System-A Review. Front. Neurol. 10, 53. 10.3389/fneur.2019.00053 30809183 PMC6380109

